# Mortality 30, 60, and 90 Days After Discharge Is Greater in Patients Who Experienced Postoperative Respiratory Depression and Pulmonary Complication

**DOI:** 10.7759/cureus.79913

**Published:** 2025-03-02

**Authors:** Robert B Raffa, Joseph V Pergolizzi, George C Dungan, Thomas L Miller

**Affiliations:** 1 Research and Development, Enalare Therapeutics, Princeton, USA; 2 Clinical Development, Enalare Therapeutics, Princeton, USA

**Keywords:** pord, postoperative pulmonary complication, postoperative respiratory depression, postsurgical mortality, ppc

## Abstract

Upon the induction of general anesthesia, a predictable sequence of physiological changes occurs within the respiratory and neuromuscular systems. The sequelae of these changes include an assortment of postoperative pulmonary complications (PPCs), including postoperative respiratory depression (PORD), that are observed during the immediate postoperative period and in the post-anesthesia care unit (PACU). These adverse events are anticipated, because several of the drugs that are used during surgery (e.g., opioids, which are traditionally used to manage pain during and after surgery), albeit therapeutically beneficial, have these adverse effects as part of their pharmacology. Nevertheless, the effects are traditionally considered transitory. However, several studies provide evidence suggesting that PPC-related morbidity and mortality extend 30, 60, and even 90 days after discharge from the hospital. These studies are summarized and assessed in this narrative review. Although exact estimates vary depending on the definitions used, the type of surgery, patient population, and risk factors (such as age), it is clear that PORD and other PPCs can be severe postoperative complications with significant associated mortality risks that extend weeks to months after discharge from the hospital.

## Introduction and background

Recovery from surgery is influenced by multiple factors, including the administration of intra- and postoperative pharmacologic medications that, albeit beneficial and designed to improve surgery outcomes, have the unwanted and undesirable side effect of producing deleterious respiratory depression (hypoventilation), too slow or too shallow breathing that leads to clinically significant hypoxia or hypercapnia [1]. Examples of such medications include those used for anesthesia (e.g., propofol, barbiturate, etomidate, and ketamine), pain relief (e.g., opioids), muscle relaxation (e.g., antagonists, depolarizing agents, and cholinesterase inhibitors), and anxiety (e.g., benzodiazepines) [2]. Not only can these medications individually produce respiratory depression, but also when co-administered during surgery, the combined effect can be additive or even synergistic [3].

Postoperative respiratory depression (PORD), aside from the obvious risk of immediate hypoxic/metabolic organ damage, is a significant contributor to postoperative pulmonary complications (PPCs), and the degree to which PORD begets PPCs is not well-defined and likely underestimated. The prolonged and delayed recovery from mechanical ventilation can induce purpose, which in turn can result in decreased compliance, poor oxygenation, subtle trauma (atelectrauma), and induction of an inflammatory cascade. These PCCs can then grow into a bigger problem.

Estimates of the prevalence of mortality following the occurrence of postoperative respiratory depression (PORD) or other PPC range from about less than 1% to more than 20% or even greater depending on the type and duration of surgery and the patients' preexisting risk factors [4-9]. Complicating estimation is the surprising lack of a consistent and agreed-upon definition of PPC or the causative PORD [6,10-18]. Nevertheless, with the large number of surgical procedures performed each year, even a small percentage of PPC translates into a very large number of affected patients. Estimates of the current annual number of procedures in the United States are about 50 million for inpatient procedures [19] and an additional approximately 20 million for outpatient procedures [20]. Also, there are epidemiologic and medical drivers for an increasing number of surgeries, such as an increasing lifespan in many countries, with the associated age-related medical conditions, general rise in global wealth, and advances in surgical technique and availability (such as remote surgery, robotic surgery, and telesurgery). Even these numbers are dwarfed by estimates by the World Health Organization (WHO) [21] and for unmet need worldwide for surgical procedures in low-income and middle-income countries [22].

## Review

Objective/purpose

The occurrence and seriousness of PPCs, including those induced by PORD, are widely recognized, and the negative consequences on patient recovery, hospital stay, and cost to the healthcare system are well-known [5,7,23-28]. There is also a known relationship between the occurrence of PPCs and underlying PORD and increased morbidity and mortality in the immediate postsurgical recovery period [13]. However, what is less well-known is that the negative sequelae of the conditions that lead to patients' in-hospital experience of PPCs caused by PORD can have negative consequences on postsurgical recovery weeks and even months after the patient has been discharged from the hospital. Studies have documented serious negative consequences, including mortality, 30, 60, and 90 days after discharge from the hospital [29]. The purpose of the present communication is to summarize the available information about long-term negative consequences of surgical interventions, including greater 30-, 60-, and 90-day mortality, and to critically assess whether there is evidence that PPCs including those induced by PORD are a causative factor of mortality during this extended period.

Methods

A literature search was conducted using databases such as PubMed®, Google Scholar, Ovid Medical Literature Analysis and Retrieval System Online (MEDLINE®) for recent publications in the English language on the topic of PORD and PPC and particularly the reported mortality 30, 60, and 90 days after discharge from the hospital. MeSH search terms such as "postoperative respiratory depression", "postoperative pulmonary complications", and "mortality" and a variety of combinations of them were used. In addition to the content itself of the identified publications, citations within them were searched. Additionally, review articles on each of the broad topics were utilized to supplement background information. The material was reviewed for applicability to the specific target topic, namely, the identification of studies that provide insight into or discuss the physiological sequelae of the intra- and perioperative administration of medications that depress respiration as adverse consequences of their use and whether the impact is underestimated by not recognizing the extension of the deleterious effects into the post-discharge period.

Results

Postoperative Pulmonary Complications and Postoperative Respiratory Depression

PPCs refer to a spectrum of respiratory issues that arise after surgery and contribute significantly to patient morbidity and mortality [30]. Some examples are as follows: atelectasis (the collapse of part or all of the lung due to the blockage of the airways or to pressure from outside the lung), pneumonia (the infection of the lungs caused by bacteria, viruses, or fungi), acute respiratory distress syndrome (severe inflammation and fluid buildup in the alveoli that leads to respiratory failure), pulmonary edema (fluid accumulation in lung tissues and alveoli, which impairs gas exchange), and bronchospasm (the constriction of the airways, leading to wheezing and difficulty breathing). The broad range of the estimates of the prevalence of PPCs vary widely for patients undergoing surgery due in part to the variable influence of risk factors, such as the type of surgery, the duration of surgery, the type of medications (e.g., opioid and benzodiazepine), the route of the administration of anesthesia, the duration of anesthesia, preexisting pulmonary conditions (e.g., asthma, chronic obstructive pulmonary disease {COPD}, and sleep apnea), advanced age, poor physical condition, obesity, a history of smoking, and the use of other sedative medications. It is also influenced by the healthcare setting and the type and extent of pre-, intra-, and postoperative prevention and management. Nevertheless, PPCs are a significant concern because they can lead to increased hospital stay and cost, morbidity, and mortality.

PORD is a condition that is characterized by inadequate respiration following surgery, resulting from the residual effects of general anesthetics, opioid analgesics, neuromuscular blockers, and other medications [8,31]. Clinical signs and symptoms of PORD include reduced respiratory rate, shallow breathing, decreased oxygen saturation (hypoxia), increased carbon dioxide levels (CO₂) (hypercapnia), and even altered mental status (e.g., confusion and drowsiness). These clinical observations cannot include underlying sequelae such as atelectasis and inflammation. However, the lack of a universally accepted definition of PORD and inconsistent criteria used among studies and in clinical guidelines have resulted in a wide disparity in the reporting of its prevalence. Disparities result from variations in the establishment of thresholds for respiratory rate, oxygen saturation, and carbon dioxide levels, as well as the clinical significance attributed to different symptoms.

Examples of criteria that have been used in studies and guidelines include the following: The first example is symptoms such as a reduced level of consciousness, difficulty in arousal, bradypnea (i.e., slow breathing), and cyanosis (the bluish discoloration of the skin and mucous membranes) as indicative of serious ongoing respiratory depression. However, these are very qualitative criteria and thus depend on the healthcare providers' experience and custom of the practice site and whether they typically identify relatively well-progressed PORD. The second example is oxygen saturation (SpO₂) level below about 90% or 92%. Despite the administration of supplemental oxygen, although a relatively common criterion, there is dispute about the precision of such measurements, and the relevance of specific levels on the broader negative effects of PORD is somewhat tenuous. The third example is hypercapnia (partial pressure of carbon dioxide {PaCO₂} greater than 45 mmHg {6 kPa}) and hypoxemia (partial pressure of oxygen {PaO₂} less than 60 mmHg {8 kPa}) based on arterial blood gas (ABG) measurements. Although the precision of such measurements is generally high, the relationship between specific levels and specific negative effects of PORD is less well-established. The fourth example is a respiratory rate of less than eight or 10 breaths per minute. However, this is thought by some to be an excessively stringent measure, suggestive of impending respiratory failure, and therefore likely underestimates the physiological harm of less drastic subnormal rates. The last example is the need for an opioid antagonist such as naloxone or the administration of respiratory stimulants to reverse sedation or respiratory depression. However, this criterion only captures impending acute respiratory failure and misses the broader physiological harm of PORD.

Although PORD is a significant clinical concern, the varying definitions lead to varying estimates of prevalence and overall clinical significance. Nevertheless, despite the lack of a universally accepted definition, recognizing and promptly addressing PORD are crucial for patient safety and for optimal postoperative care.

That the prevalence of PORD is likely underreported was the topic of the review by Ayad et al. [32]. The study reviews the characterization and monitoring of PORD, with a focus on opioid-induced respiratory depression, which is a significant concern in the perioperative setting. The review emphasizes that existing methods for detecting and monitoring PORD, such as the commonly used methods of intermittent pulse oximetry and capnography, have serious limitations and often fail to identify patients experiencing PORD, particularly those with subtle or intermittent episodes of PORD. As a result, existing monitoring contributes to underdiagnosis and delayed intervention/treatment of PORD. The authors advocate for the use of new technologies and strategies that continuously monitor and integrate multiple physiological parameters, such as spirometry including tidal volumes, oxygen saturation, and continuous end-tidal CO₂, in conjunction with a more comprehensive definition set for respiratory sufficiency. The findings underscore the critical need for improved education and awareness among healthcare providers regarding the risks and management of PORD.

Within-Hospital Mortality

PPCs, including those induced by PORD, occur in a significant number of patients undergoing major surgery, with the incidence varying widely depending on the type of surgery and patient risk factors. While the exact mortality rate is not well-defined, severe cases can lead to critical outcomes including death. The American Society of Anesthesiologists (ASA) suggests that respiratory depression is a leading cause of preventable death in the postoperative period.

Three well-controlled studies serve as examples that demonstrate that the true prevalence of PPCs is closer to the upper end of the early historical estimates, even when the criteria for the outcome measures are quite stringent.

**Figure 1:**
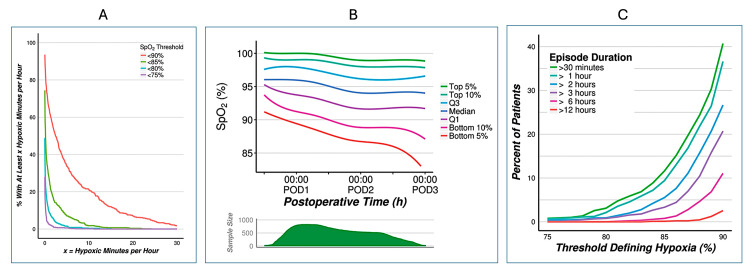
Within-hospital mortality (A) Raw oxygen saturation (SpO₂) data: the incidence of patients with an average number of minutes per hour in hypoxemia > X during monitoring, according to progressive SpO₂ thresholds characterizing hypoxemia. (B) Raw SpO₂ data: the distribution of SpO₂ across the patients in the sample, over postoperative time (POD, postoperative day; Q, quartile). Curves estimated using quantile regression with restricted cubic splines. (C) Smoothed data: the incidence of at least one single hypoxic episode of varying minimal duration under progressive thresholds characterizing hypoxemia. Source: Sun et al. (2015), with permission [8]

The first example is the study by Sun et al. [8]. This well-designed and controlled study aimed to determine the incidence and duration of postoperative hypoxemia in a diverse surgical population. It was a prospective, blinded, observational study of more than 800 patients in a large tertiary care hospital of adult patients undergoing noncardiac surgery. Continuous pulse oximetry was used to monitor oxygen saturation (SpO₂) levels for up to 48 hours post-surgery. The clinicians, as well as the patients, were blinded to the oximetry data in order to avoid influencing clinical decisions based on the monitoring results. The study found that 37% of patients experienced at least one episode of hypoxemia (defined as SpO₂ < 90%) during the first 48 hours after surgery, and 8% experienced at least one episode when defined as SpO₂ < 85% (Figure 1A). Individual patients fared worse (Figure 1B). Hypoxemia was not only common but also often persistent, with many patients experiencing prolonged or repeated episodes. Around 21% of patients had hypoxemia that lasted more than 10 minutes, indicating sustained low oxygen levels, and 10% of patients had hypoxemia lasting longer than 30 minutes (Figure 1C).

The prolonged periods of low oxygen saturation often went unnoticed by the hospital care team. The study concluded that postoperative hypoxemia is both common and persistent, often occurring without being detected in routine clinical practice.

**Figure 2:**
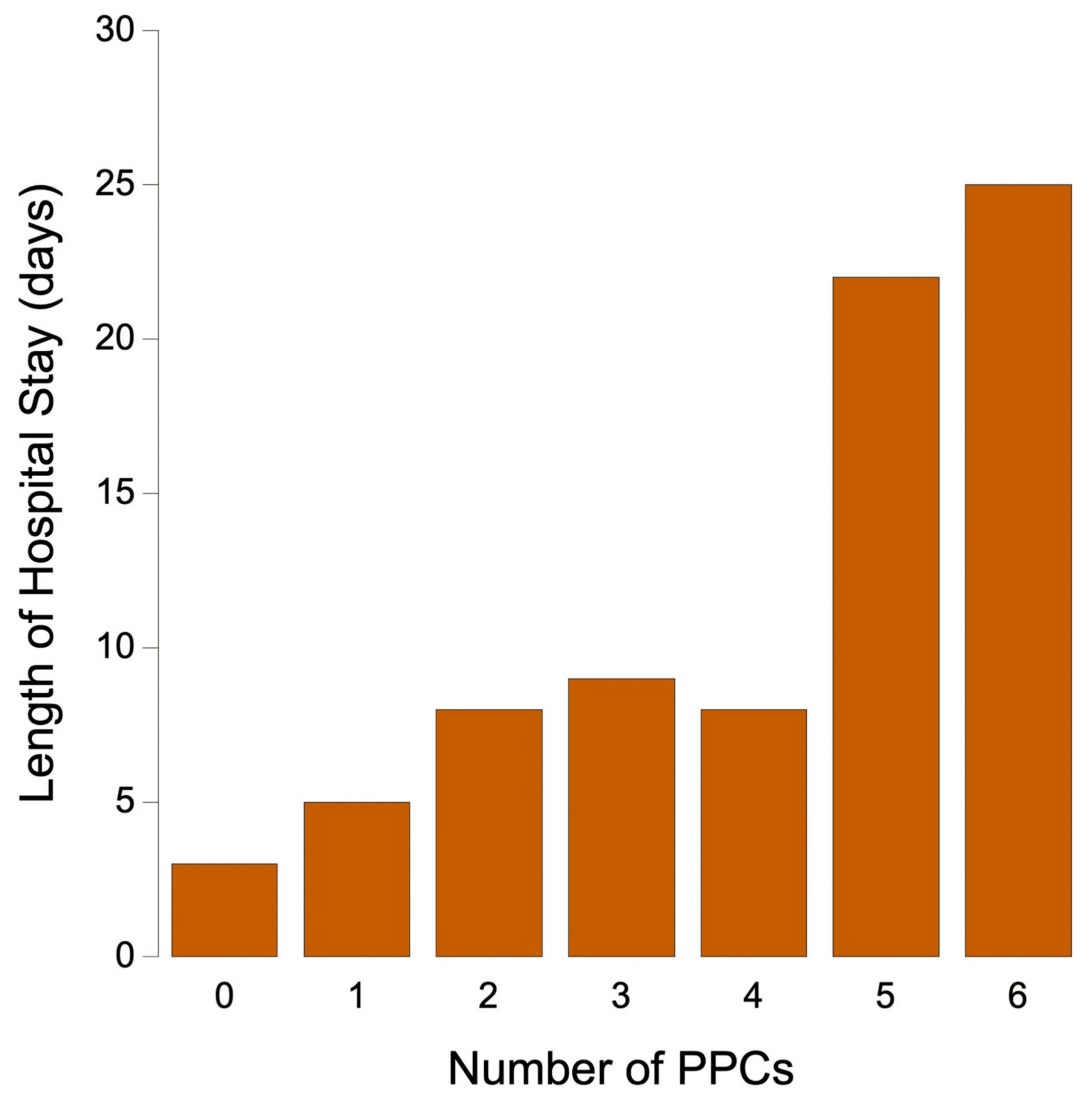
Hospital stay as a function of the number of PPCs The length of hospital stay as a function of the number of postoperative pulmonary complications (PPCs) experienced in a multicenter, prospective, observational study of 1,202 physical status 3 noncardiothoracic surgical (mostly abdominal, orthopedic, and neurological) patients in seven US academic institutions. Plot created from data by Fernandez-Bustamante et al. [13]

The second example is the study by Fernandez-Bustamante et al. [13]. The study involved 1,202 patients, predominantly undergoing abdominal, orthopedic, and neurological surgeries. Noncardiothoracic surgeries were excluded, because these are known to be associated with greater occurrences of PPCs. Despite excluding the cardiothoracic surgical patients, the study found that 33.4% of the patients experienced at least one PPC. This study is noteworthy for also collecting data on longer-term outcomes. Patients who had experienced PPCs while in the hospital had a significantly higher early postoperative mortality rate, increased likelihood of intensive care unit (ICU) admission, and longer ICU and hospital stays (Figure 2). Even mild PPCs were associated with these greater adverse outcomes.

**Figure 3:**
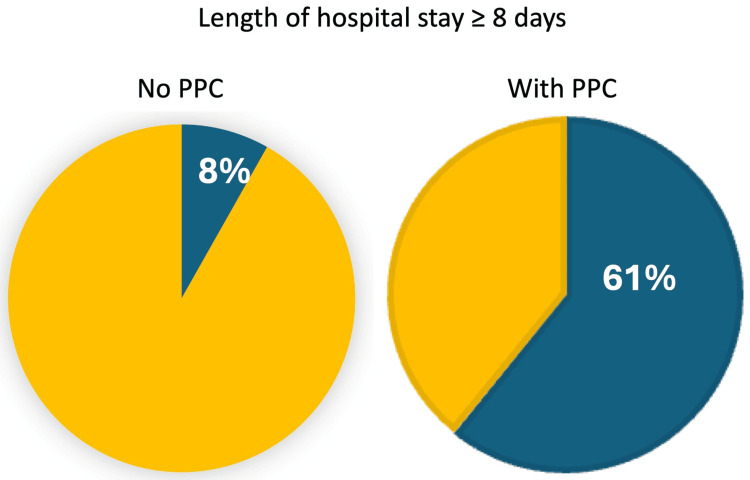
Prolonged hospital stay with one or more PPCs Comparison of prolonged length of hospital stay (≥8 days) of surgical patients (open reduction and internal fixation of vertebral fractures) who experienced no, or at least one, postoperative pulmonary complication (PPC). Plot created from data by Ye et al. [33]

The effect of experiencing PPC on the length of hospital stay was also reported by Ye et al. in a retrospective study using the American College of Surgeons National Surgical Quality Improvement Program database of 900 adult patients after open reduction and internal fixation of vertebral fractures [33]. Only 6% of patients who did not experience a PPC had a hospital stay of eight or more days, whereas 61% of patients who had experienced a PPC had a stay of eight or more days (Figure 3).

**Figure 4:**
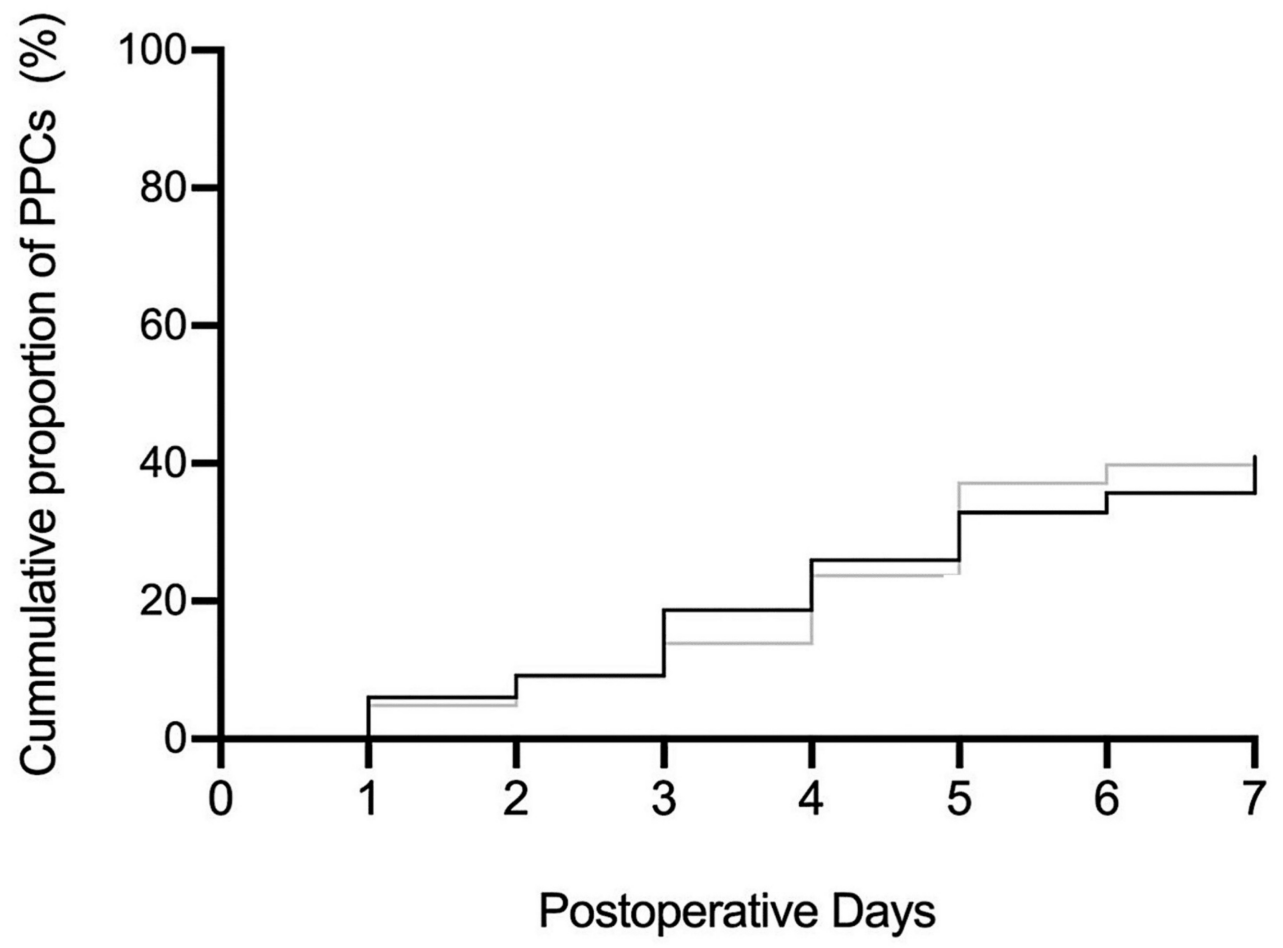
Development of PPCs over a week following surgery The Kaplan-Meier curve of the development of postoperative pulmonary complications (PPCs) over the week following on-pump cardiac surgery in a prospective randomized controlled trial of 694 patients. Source: Li et al., with permission [34]

The third example is the study by Li et al. [34]. This study investigated the comparative effects of driving pressure-guided ventilation versus conventional mechanical ventilation on pulmonary complications in patients undergoing on-pump cardiac surgery. The study was a randomized clinical trial involving 694 patients, divided into two groups: one receiving driving pressure-guided ventilation and the other receiving conventional ventilation. A key finding relevant to the current review is that PPCs occurred in 40.3% of patients in the driving pressure-guided group and 40.9% in the conventional group. This finding is another example that well-controlled studies reveal a prevalence of PPCs nearer to, or exceeding, the high end of the historical studies. It also demonstrates the continued development of PPCs over the seven days postoperatively (Figure 4).

Can PORD Be Prevented by Simply Administering Oxygen?

It might seem logical that the occurrence or severity of PORD could be reduced by the administration of high concentrations of oxygen during the perioperative period. However, while oxygen is a critical element in perioperative care to prevent hypoxia and ensure adequate tissue oxygenation, there is evidence that high concentrations of oxygen can be harmful, leading to oxygen toxicity and related complications. For example, elevated oxygen levels increase the production of reactive oxygen species (ROS). When the production of ROS exceeds the body's antioxidant defenses, it leads to oxidative stress, causing cell and tissue injuries involving damage and death to cell components such as lipids, proteins, and DNA. Lumb and Walton review various studies that highlight the negative outcomes of the use of high oxygen levels for surgical patients [35]. The evidence suggests that the use of high-concentration oxygen in the perioperative setting can lead to worse outcomes, including higher rates of postoperative (including pulmonary) complications and mortality.

Can PORD and Subsequent PPCs Be Avoided by Administering Opioids by an Alternate Route?

Since the use of opioid pain relievers during the perioperative period, usually administered systemically, is a known major risk factor for PORD, could simply administering the opioid by a non-systemic route reduce the prevalence of PORD and perhaps PPCs? A partial answer to this question is addressed in a review by Pöpping et al. [36]. The review investigated the impact of analgesia administration by the epidural, rather than systemic, route on postoperative outcomes, focusing on mortality and morbidity. Epidural analgesia involves the injection of anesthetics and/or analgesics into the epidural space of the spinal cord. This technique is believed to offer better pain control, reduce opioid consumption, and potentially enhance recovery, but its effects on long-term outcomes such as mortality and overall morbidity remain a topic of research. The study by Pöpping et al. was a systematic review and meta-analysis of randomized controlled trials involving patients undergoing major surgeries who were administered epidural analgesia versus those receiving alternative analgesic methods [36]. The meta-analysis found no significant difference in overall mortality between patients receiving epidural analgesia and those receiving other forms of analgesia. However, better pain relief was consistently reported in the epidural analgesia group compared to those receiving systemic opioids or other analgesic techniques. In addition, there was a significant reduction in the incidence of postoperative complications, including respiratory complications in patients receiving epidural analgesia. As a result, patients receiving epidural analgesia often had a shorter length of hospital stay, likely due to better pain management and reduced complications. The article concludes that epidural analgesia does not significantly reduce overall mortality, but it offers substantial benefits in reducing postoperative morbidity, particularly cardiovascular, respiratory, and infectious complications. The finding supports the use of epidural analgesia for improved postoperative outcomes, especially in high-risk patients and major surgeries, particularly for thoracic, abdominal, and lower extremity surgeries, where it can improve patient satisfaction and health-related quality of life postoperatively.

However, since the era of Pöpping et al.'s study, which was published in 2014, recent studies suggest that the benefits of the use of epidural analgesia for managing postoperative pain unfortunately might not be as substantial as previously believed [36]. Evolving evidence has prompted a re-evaluation of its overall efficacy and safety. In short, despite its benefits, it is now recognized that epidural analgesia also carries significant risks [37]. These include the potential for serious complications such as epidural hematoma, infection, and neurological injury. Additionally, epidural analgesia may contribute to hypotension, urinary retention, and motor block, which can impede early mobilization and recovery. Alternatives such as patient-controlled analgesia (PCA) and other multimodal analgesia techniques are increasingly being recognized for their effectiveness in providing adequate pain relief with potentially fewer risks. These methods may offer comparable analgesic efficacy while reducing complications, thus challenging the role of epidural analgesia as a default choice for postoperative pain management. Hence, while the non-systemic (epidural) route decreases some postoperative adverse effects, it introduces some others. Therefore, although the non-systemic route is a potentially valuable tool to reduce PORD and subsequent PPCs, it should not be universally applied without considering alternative options and individual patient needs. The recent move toward personalized pain management strategies reflects a broader trend in healthcare aimed at optimizing outcomes and minimizing risks.

Delayed (Post Hospital Discharge) Mortality

The proposal that deaths that occur 30, 60, or even more days after surgery and after discharge from the hospital might be attributable to in-hospital PORD or other PPC is relatively new. The lack of attribution until recently seems understandable since the hospitals might not uniformly follow patients that long after discharge, and they might not be anxious to admit the connection. Likewise, the healthcare provider treating the patient at the time of death might not associate the mortality with the adverse effects of a surgery that occurred weeks or even months prior. Itemized below are some succinct summaries of findings regarding delayed post-discharge mortality, arranged by post-surgery length and type of surgery.

Thirty-day mortality: The mortality rate within 30 days following surgery varies widely, similar to in-hospital mortality, and depends on several factors, including the type of surgery, the patient's risk factors, overall health, and the presence of comorbid conditions. Examples of estimates based on the type of surgery are as follows: The 30-day mortality rate for general surgical procedures is about 1%-6%, depending on the complexity and invasiveness of the procedure and demographics [38,39]; for high-risk procedures such as coronary artery bypass grafting (CABG) and heart valve surgeries, the 30-day mortality rate can range from 2% to 5% [40,41]; major oncologic surgeries also show varying 30-day mortality rates, generally around up to about 7%, depending on the cancer type and stage at the time of surgery [42]; hip fracture surgeries in elderly patients have a 30-day mortality rate of approximately 6%-13%, reflecting the generally greater frailty and higher risk profile of this patient population [43,44]; and patients undergoing emergency surgeries typically have higher 30-day mortality rates, often exceeding 10%, especially in the elderly or those with significant comorbidities [45].

Sixty-day mortality: The 60-day postoperative mortality rate provides an intermediate view of delayed outcomes, bridging the gap between the immediate postoperative period and longer-term assessment. This timeframe can capture additional complications that may arise after the initial recovery phase but before long-term outcomes are fully developed or apparent. Examples of estimates based on the type of surgery are as follows: The 60-day mortality rate for general surgical procedures is up to 10% for high-risk patients [46]; for cardiac surgeries such as CABG and heart valve replacement, the 60-day mortality rate can range from 3% to 7%, reflecting ongoing risks from both the surgery and the patient's cardiovascular condition [41]; the 60-day mortality rate after major oncologic surgeries varies, typically around 3%-11%, depending on the type and stage of cancer, as well as the patient's condition [47]; for hip fracture surgeries in elderly patients, the 60-day mortality rate is approximately 8%-12%, reflecting the generally greater frailty of this population and the significant impact of such injuries on mobility [48]; and emergency surgeries continue to show higher mortality rates at 60 days, often around 11%-18%, especially in older patients and those with multiple comorbidities [49].

These estimates highlight the importance of ongoing patient monitoring and intervention even during the 60-day postoperative period, which is critical for addressing complications that may arise beyond the immediate recovery phase.

Ninety-day mortality: As with the in-hospital and shorter post-hospital-discharge time periods, the 90-day postoperative mortality rate also varies widely depending on the type of surgery, patient demographics, and underlying health conditions. Examples using the same types of surgeries as above are as follows: The 90-day mortality rate for general surgeries tends to be higher than the 30-day rate and similar to the 60-day rate, often ranging from 2% to 6%, depending on the complexity of the procedure and patient factors [50]; for major cardiac procedures such as CABG and heart valve surgeries, the 90-day mortality rate can range from 4% to 8%, reflecting the long-term risks associated with these extensive procedures [41]; the 90-day mortality rate after major cancer surgeries varies but is typically around 4%-8%, depending on the type of cancer and stage at the time of surgery [51]; the 90-day mortality rate for hip fracture surgeries in elderly patients can range from 3% to 8%, reflecting generally higher-risk nature of this population [52]; and emergency surgeries continue to have higher mortality rates at 90 days, up to nearly 20% among elderly patients and those with significant comorbidities [50].

These examples illustrate the increased risk of patient mortality over a surprisingly long postoperative period (three months) and underscore the importance of an awareness of the need for extended diligence and the continuous monitoring and management of patients after surgery, especially those with higher-risk profiles.

Of course, the important question is what fraction of these delayed deaths are related to an in-hospital experience of a PPC. This question is addressed subsequently.

Is Age the Simple Explanation for Delayed (Post Hospital Discharge) Mortality?

Perhaps because patients 65 years or older comprise a significant portion of surgical procedures, there might be some inherent greater age-related susceptibility that artificially inflates the postoperative morbidity and mortality data. In other words, perhaps, these patients are frail and undergoing life-extending rather than curative surgeries, and a certain percent of mortality during the post-hospitalization time might be anticipated, independent of experiencing a PPC. This was a question investigated by Plantz et al. in an analysis of more than 17,000 patients in an American College of Surgeons surgical outcome database that found increased rates of 30-day postoperative readmissions and medical complications among patients aged 65 and older following arthroscopic rotator cuff repair (ARCR) [53]. The study utilized data from the American College of Surgeons National Surgical Quality Improvement Program database, encompassing a large cohort of patients who underwent ARCR. Multivariate logistic regression models were used to analyze the data and identify independent predictors of readmissions and complications. The study found that patients aged 65 years and older had a significantly higher rate of 30-day unplanned readmissions compared to their younger counterparts. Specifically, older patients experienced an increased incidence of medical complications, including respiratory complications. Importantly, however, the older patients did not have a higher 30-day mortality. Therefore, it appears that a cynical explanation that older 30-, 60-, or 90-day post-discharge patients might have died anyway during this period is not valid.

Is the 30-, 60-, and 90-Day Post-discharge Mortality Related to PORD/PPCs?

Connection based on physiological sequelae: Miskovic and Lumb address the question of a physiological nexus and chain of events that lead from the initial induction of anesthesia, through the occurrence of PPCs, to delayed post-hospital-discharge morbidity and mortality [29].

Adverse effects of general anesthesia on the respiratory system start as soon as the patient loses consciousness [54]. The central respiratory drive is depressed, which results in periods of apnea, followed by a return of spontaneous respiration with a dose-related reduction in minute ventilation (the volume of gas inhaled or exhaled by the lungs per minute). Critically, the usual ventilatory responses to hypoxia and hypercapnia are blunted by anesthetics, even at low doses [55]. In addition to the negative effects on the lungs and brainstem nuclei, respiratory muscle function declines immediately after the induction of anesthesia, leading to a reduction of functional residual capacity (FRC) of 15%-20%, whether or not the patient is administered a neuromuscular-blocking drug [54].

These and other changes in the physiological control of normal respiration and response to diversions from homeostatic control lead to PORD, which then may lead to PPCs [2]. A particularly common and important PPC is the development of atelectasis, which is the collapse of part or all of the lung when the lung sacs (alveoli) cannot inflate properly. The result is not only a loss of effective lung volume (surface area) for gas exchange but also a change in how mechanical force for lung expansion with each breath is now unevenly applied to the alveolar walls, resulting in tissue damage (atelectrauma). Atelectasis occurs in more than 75% of surgical patients who are administered general anesthesia with a neuromuscular blocker [56]. Such changes can be observed on a computed tomography (CT) scan to begin a few minutes after the induction of anesthesia [10]. The early onset of the physiological changes that occur during the start of the surgical procedure (the induction of anesthesia) is amplified by the concurrent administration of drugs that depress respiration (such as opioids, benzodiazepines, and propofol) at central (e.g., brainstem nuclei such as the pre-Bötzinger complex) or peripheral (e.g., airway muscles, abdominal muscles, diaphragm, and carotid bodies) sites.

Once the patient is moved to the post-anesthesia care unit (PACU), hypoxia associated with respiratory depression is common and is considered by some to be a PPC [26]. The presentation of respiratory depression using the noninvasive measurement of blood oxygen may be substantially blunted in the presence of the application of supplemental oxygen. Atelectasis usually resolves in a few hours after minor surgery but persists after major surgery, particularly if there is a residual influence of a neuromuscular-blocking drug [57]. However, trauma and inflammation may remain active, affecting lung mechanics and oxygen diffusion, and potentially propagate. Episodes of hypoxemia continue to be common after discharge from the PACU, and it can take days for the normal alveolar-to-arterial oxygen difference to reestablish. Atelectasis has been reported in a review of a heterogeneous cohort of non-thoracic patients to be present (by radiological evidence) in 57% of the patients, with an increasing trend over postoperative days 1, 2, and 3 [58].

The anesthetic agents, neuromuscular-blocking drugs, postoperative analgesic drugs (particularly opioids), residual pain, disturbed sleep, and the inflammatory response to surgery continue to persist, as evidenced by the fact that peak expiratory flow rate and lung function tests are all reduced significantly after surgery [59]. The normal activity of most respiratory muscle groups is impaired after major surgery, beyond simple muscle weakness, also involving poor coordination between muscle groups and the failure of the normal physiological reflexes and control mechanisms on which their activity depends [60]. In fact, respiratory control may continue to be subnormal, including reduced ventilatory responses to hypercapnia and hypoxia, for extended periods. In one study, responses were still measurably impaired six weeks after surgery [61]. This suggests that there is some functional physiological plasticity in the respiratory control mechanisms at the time of surgery that takes some time to return to normal homeostasis.

As summarized in the review by Miskovic and Lumb, "This combination of reduced FRC, residual atelectasis, an ineffective cough, and abnormal respiratory control, forms an ideal situation for PPCs to develop" [29]. Based on the findings of the current review, it can be added that the problems caused by this combination persist into the post-hospital-discharge period.

Connection based on clinical evidence: An excellent extensive review of the major prospective and retrospective studies between 2000 and 2015 that evaluated PPCs is provided by Miskovic and Lumb [29]. Nineteen studies are included. To quote from the review, "Mortality is increased in both the short and long term in patients who develop a PPC. One in five patients (14%-30%) who have a PPC will die within 30 days of major surgery compared with 0.2%-3% without a PPC" [6,7,25,39,62,63]. "The 90-day mortality has been shown to be significantly increased in those with a PPC: 24.4% versus 1.2%" [64]. "An observational study of two large databases shows long-term significant differences in mortality rates with and without PPCs: 45.9% versus 8.7% at one year or 71.4% versus 41.1% at five years" [39].

**Figure 5:**
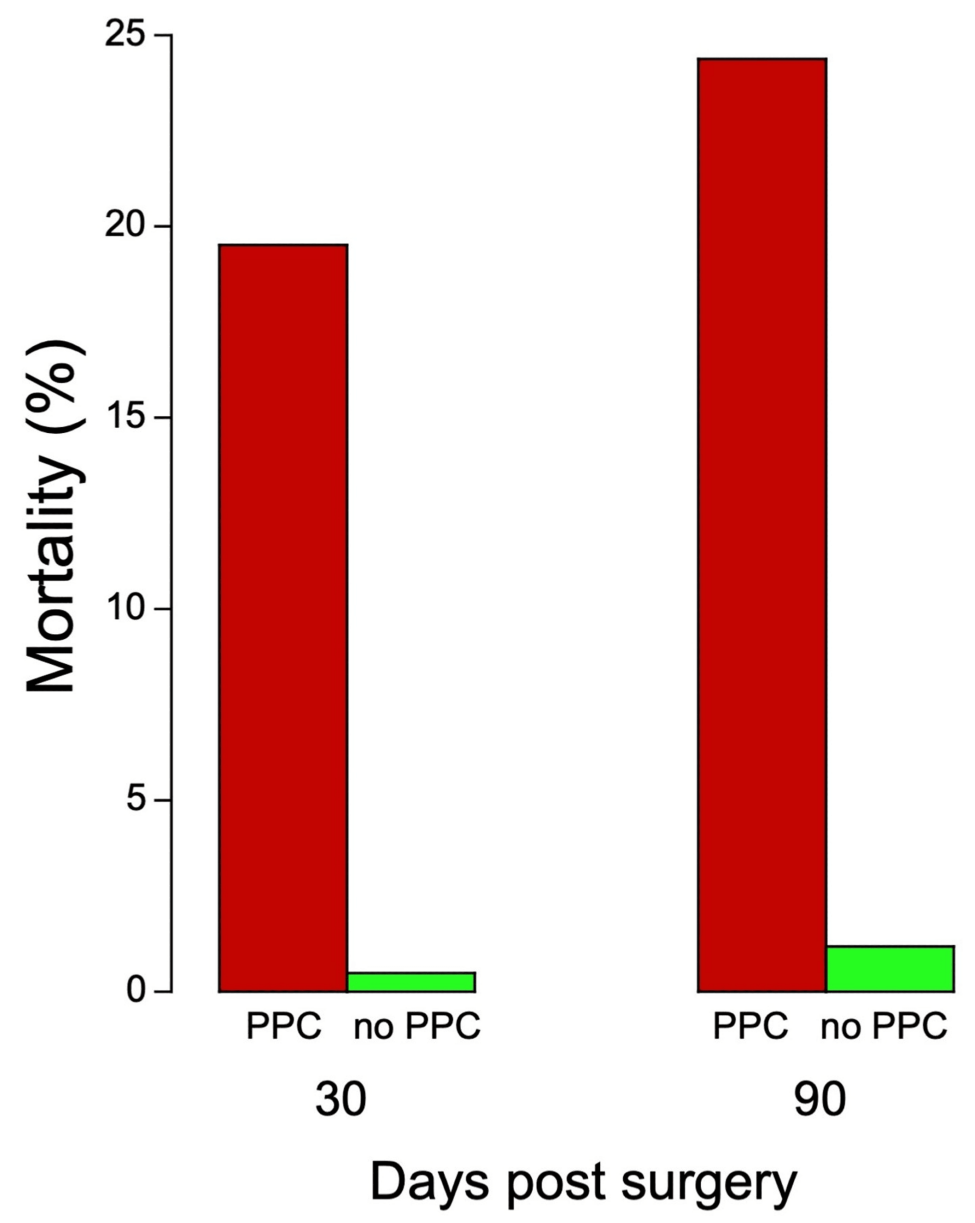
Thirty- and 90-day mortality post-surgery Thirty- and 90-day mortality in a prospective, multicenter study of 2,464 patients in 59 community, intermediate referral, and major tertiary care facilities in large-city and rural hospitals in Spain covering a wide range of scheduled or emergency surgical procedures under general, neuraxial, or regional anesthesia. Created from data by Canet et al. [64] PPC: postoperative pulmonary complication

The study by Canet et al. is particularly noteworthy because of the extensive controls [64]. The study involved a large, diverse cohort to address the limitations of previous studies that often included narrow patient demographics and surgery types. It was a prospective, multicenter, observational study of a random sample cohort of patients who underwent nonobstetric in-hospital surgical procedures. It included patients from 59 large-city and rural hospitals in Spain (including community, intermediate referral, and major tertiary care facilities), covering a wide range of scheduled or emergency surgical procedures under general, neuraxial, or regional anesthesia. The cohort consisted of 2,464 patients, randomly selected to reflect the general surgical population. To control for the seasonal, weekly, and daily distribution of site caseload, each center was notified of seven randomly assigned days of the year, one for each day of the week. For the 20 highest-volume centers, an interval of at least 15 days between sampling days was required. The main outcome measured was the occurrence of PPCs, which included respiratory infections, respiratory failure, bronchospasm, atelectasis, pleural effusion, pneumothorax, and aspiration pneumonitis. The 30-day mortality rate was found to be significantly higher in those patients who experienced PPCs (19.5%) than in those who did not (0.5%). Likewise, the 90-day mortality rate was found to be significantly higher in those patients who experienced PPCs (24.4%) than in those who did not (1.2%) (Figure 5). Given the extensive controls used in the study, the results are suggestive of causality between the in-hospital experience of PPC and greater mortality 30 and 90 days after discharge from the hospital.

Discussion

PPCs are known to be associated with a deleterious effect on mortality following surgery. The institution of the PACU (in the form of a recovery room) was largely to improve outcomes, with substantial emphasis on the assessment of the patient for evidence of cyanosis, stridor, and asphyxiation [65]. These dedicated rooms were more widely established after the increased use of an anesthetic agent. Thus, the importance of pulmonary complications associated with anesthesia has been understood for decades. However, the current review suggests that the current monitoring or interventional approaches may be insufficient.

Data is amassing, which demonstrates that mortality resulting from PPCs not only is higher than acceptable but also can occur at least 90 days after a procedure. While we can predict the vulnerable patients, for example, frailties of age and comorbidity, we cannot control who needs surgery. Therefore, the process needs to adapt to address these high mortality rates. PPCs need to be recognized early and treated aggressively.

PORD is a significant contributor to PPCs that are highly prevalent, well-documented, likely underestimated, and not well-defined and controlled. PORD is not just a transient slow-breathing rate. PORD represents a complicated emergence of breathing control, at a time when lung expansion is heterogeneous (including atelectasis) and requires re-recruitment to abate building trauma and inflammation. The goal is not to put the patient back on a ventilator, which contributes to atelectasis, barotrauma, and inflammation, nor should it be to treat a ventilation problem with more oxygen. Instead, the emphasis should be on restoring lung recruitment and fostering spontaneous breathing control.

Current ubiquitous monitoring may be able to provide better insight into the development of PORD, prior to a cascade leading to PPC. However, the harmonization of appropriate thresholds across critical care settings should be considered, as the application of some proposed vital sign monitoring thresholds may be inappropriate barriers to early identification. What constitutes signs of respiratory depression in a medical ICU may well be much more sensitive than the thresholds used in the context of post-anesthesia emergence. This may be due to the rather severe nature of the interventions currently available for the emergent management of PORD.

## Conclusions

Although exact estimates vary depending on the definitions used and there is variation due to the type of surgery, patient population, and risk factors (such as age), it is clear that PORD can lead to serious postoperative complications associated with significantly longer hospital stays and increased in-hospital mortality risk. Less known is that there are reports that PORD and PPCs have an increasingly well-documented impact on mortality rates that extend weeks to months after discharge from the hospital. That this connection is not more widely recognized may be due in part to issues with the continuity of care during the transition of patients from the hospital to the care of their community healthcare providers. Mitigating PORD- and PPC-associated delayed (post-discharge) morbidity and mortality may comprise preventive pre- and perioperative strategies, careful and accurate monitoring both in-hospital and post-discharge, and the increased recognition of the potential for delayed serious consequences.
